# Considerations in referring older patients to hospital in acute situations: a qualitative study based on the experiences of healthcare providers

**DOI:** 10.1186/s12875-026-03453-9

**Published:** 2026-07-20

**Authors:** Cindy Cardol, Britt Wagenaar, Hanneke Merten, Yvonne de Man, Jeroen Hasselaar, Linda van Eikenhorst, Cordula Wagner

**Affiliations:** 1 Nivel, Otterstraat 118, 3513 CR Utrecht, the Netherlands; 2 Department of Primary Care, Radboud University Medical Centre Nijmegen, Nijmegen, the Netherlands; 3 Department of Public and Occupational Health, Amsterdam UMC, Vrije Universiteit Amsterdam, Van Der Boechorststraat 7, 1081 BT Amsterdam, the Netherlands; 4 Amsterdam Public Health, Quality of Care, Amsterdam, The Netherlands

**Keywords:** Hospital Referral, Patient admission, Decision making, Focus groups, Qualitative research, Palliative Care, Frailty, COPD, Hip fractures, Aged

## Abstract

**Background:**

Hospital referral in acute situations is a complex process with a significant impact on older adults. This study aims to explore the considerations of healthcare providers from both primary and secondary care when deciding whether or not to refer an older patient to the hospital in acute situations. By including perspectives of different health care providers, this study seeks to provide a more comprehensive understanding of the decision-making process.

**Methods:**

In February 2025, three online semi-structured focus group discussions were conducted with 16 primary and secondary healthcare providers. Three case studies (two hip fractures, one COPD exacerbation) guided the discussion. Data were analysed using a combination of inductive and deductive thematic analysis and were iteratively clustered and visualized around four key consideration factors.

**Results:**

Twenty subthemes were identified and mapped onto four overarching main themes, corresponding to the four key consideration factors: patient-related (e.g. network, quality of life, patient and family preferences, emotional status and living situation), healthcare provider-related (e.g. professional attitude, collaboration and familiarity with patients), case-specific (e.g. age, medical history, activities of daily living (ADL) and mobility, cognitive functioning, frailty, prognosis, the acuity of situation, alternative treatment, and risk of complications) and structural factors (e.g. ability to arrange appropriate care and the patients’ increasing care demand).

**Conclusions:**

Decision-making in hospital referrals is not determined solely by the acute condition, but is a complex and dynamic process. This highlights the importance of strengthening interprofessional communication and collaboration, addressing the shortages in the availability of appropriate patient care, and extending advance care planning (ACP) practices by involving family members to support more patient-centred referral decisions.

**Supplementary Information:**

The online version contains supplementary material available at https://doi.org/10.1186/s12875-026-03453-9.

## Introduction

A referral decision for older patients from primary to secondary care is a critical and complex process [1, 2] that, if not made adequately, may be associated with inappropriate care and an increased risk of adverse events [3–5]. Often, trade-offs must be made between four key consideration factors, namely patient, general practitioner (GP), structural and case-specific factors [6–8]. Patient related factors include for example, age, vitality, socioeconomic status and level of co-morbidity, as well as patients’ expectations, needs, preferences and values influencing referral decisions [8–10]. Factors related to GPs and other healthcare providers encompass factors such as age, gender [10], risk aversion, personality, knowledge, interests, relations with patients and colleagues, the ability to handle uncertainty [8], concerns, feelings and attitudes [11]. Furthermore, structural healthcare-related factors include waiting lists, practice organization, and proximity to the hospital [8]. Finally, case-specific factors involve the nature of the condition, severity of illness, level of acuity and whether a situation is expected or not [8–10]. Hence, referral decisions are a complex interplay between various (non)-medical factors.

Referral decisions for older and palliative patients are even more complex and patient preferences (regarding comfort and quality of life rather than curative treatments) play a more significant role. Several initiatives have been developed to address this, such as advance care planning (ACP) [12, 13] and shared decision-making (SDM) [14]. The decision to refer or not becomes even more pronounced in acute situations, where patient preferences may have changed, time constraints call for rapid judgments, and the referral decision can have significant impact. A distinction can be made between acute situations and unexpected acute deterioration of disease. In acute situations, a condition arises suddenly and the consequences of the situation depend heavily on the taken course of action. An example is a hip fracture in (frail) older adults, where the decision for a surgical intervention may signal a transition into the palliative phase [15]. In contrast, situations with acute deterioration of disease are related to chronic progressive conditions in which it is likely that an acute situation will eventually occur. For instance, older patients with Chronic Obstructive Pulmonary Disease (COPD) who experience exacerbations requiring hospital admission, making it important to address in advance whether hospitalization remains desirable for future occurrences [16–19].

Currently, in the Netherlands the responsibility for referral decisions of acute hospital care mainly rests with GPs. However, other primary and secondary healthcare providers can also play important roles in the referral process. Each healthcare provider or informal caregiver brings their own background, knowledge, experience, and perspective, which may lead to different considerations and decisions. For instance, nurses tend to adopt a more holistic view of the patient, as patients often share different information with nurses than they do with physicians [20, 21]. Medical specialists have more in-depth, disease-specific expertise regarding diagnostic and treatment options. Additionally, informal caregivers may hold other perspectives focusing on the personal situation and preferences of the person. To achieve a holistic perspective and make well-informed diagnostic and treatment decisions in line with personal needs and preferences, it is essential to integrate these viewpoints to optimize patient referrals. Achieving this requires involvement of stakeholders and timely exchange of comprehensive patient information, although the timing and completeness of this information may vary depending on the condition [22–24].

Therefore, this study aims to explore the considerations of primary and secondary healthcare providers when deciding whether or not to refer older patients to the hospital in acute situations. By including different perspectives of healthcare providers, this study seeks to provide a comprehensive understanding of the decision-making process.

## Methods

### Study design

A qualitative design was employed, using semi-structured online focus group discussions to explore healthcare providers’ perspectives on referral decisions. Three online focus groups were conducted in February 2025, involving a mix of healthcare providers from both primary and secondary healthcare settings. The group format facilitated interaction between participants, enabling the identification of both shared and differing perspectives on referral decisions [25]. This study was reported following the Consolidated Criteria for Reporting Qualitative Research (COREQ) guidelines [26] (see Additional File 1).

### Participant selection

Eligible participants for this study were Dutch-speaking healthcare providers with experience in primary (e.g. GP, community nurse, primary care nurse) or secondary care, defined here as hospital-based healthcare providers (e.g., resident physician, medical specialist, hospital nurse, nurse practitioner, or physician assistant). Participants were required to have experience in caring for patients with hip fracture or COPD. Experience was defined as direct involvement in the clinical care, management, or referral decision-making in daily practice for these patient groups.

Between December 2024 and February 2025, participants were recruited using purposive and snowball sampling to ensure maximum variation in health care providers backgrounds [27, 28]. Multiple open and personal channels were used during the recruitment process, including LinkedIn, and direct one-to-one approaches within the personal and professional network. Eligible participants were informed about the study's purpose and practical information about the focus group procedure and were given the opportunity to decide whether or not to participate.

### Data collection

Three focus group discussions were conducted via Microsoft Teams; each lasted approximately 90 min and was audio-visually recorded. The first researcher (CC) moderated all focus group sessions, while the second researcher (BW) took field notes [27]. Both researchers had some experience with qualitative research approaches, but no prior experience with conducting focus groups. To ensure quality, a senior qualitative researcher (HM) attended the first session to provide feedback, and the team engaged in reflexive discussions on their assumptions and interpretations [28].

The discussions were structured around three case study examples, including two unexpected acute cases (hip fracture) and one expected acute case (exacerbation of COPD) (see Table 1 and Additional File 2). The two hip fracture case studies were based on patient descriptions from the 2019 Dutch Adverse Events Monitor [29] and the COPD case was based on experiences of healthcare professionals from a previous pilot study [9]. All three cases were piloted in a focus group with GPs, which served as the basis of this study [9]. Drawing on the prior focus group findings, the questions were revised to reflect this study’s broader perspective of healthcare providers beyond general practitioners. For each case, three key questions were included in the topic guide, along with additional prompting questions (see Table 2 and Additional File 3). These key questions covered main themes of referral considerations, information needs, and patient preferences. Each case was presented, after which the key questions were shared in the Microsoft Teams chat to allow time for individual reflection on their considerations. Afterwards, g the three key questions were discussed in the group.

**Table 1:** Brief descriptions of the three cases for the focus groups

Case 1: Hip fracture	
A 93-year-old man fell during the night. Before the fall, he was able to stand independently, but now cannot due to severe pain. The patient has a medical history of peripheral arterial disease and coronary artery bypass grafting (2004). He is a widower and lives independently, performing activities of daily living (ADL) on his own. The general practitioner finds a painful, alert and adequate patient with normal vital signs. A diagnosis of a hip fracture is made.	
Case 2: Hip fracture	
A 90-year-old woman has fallen and can no longer get up and has severe pain. The patient has a medical history with Alzheimer’s disease and diabetes mellitus. The dementia case manager is closely involved, because a lot rests on her husband’s shoulders. In particular, the toilet visits and night-restlessness. Her husband does not want his wife to be admitted to a nursing home. The general practitioner suspects a hip fracture.	
Case 3: Exacerbation of COPD	
A 78-year-old man with severe COPD has shortness of breath, his inhalation medication or increasing the oxygen does not help. The patient has a medical history of COPD GOLD-IV, diabetes mellitus, myocardial infarction (2010) and chronic heart failure. His regular pulmonologist has indicated that there are no more therapeutic options that the hospital can offer him. The patient indicated that he does not want to go to the hospital but his children are quite urgent in their request for admission. The general practitioner suspects an exacerbation of COPD.	

**Table 2:** Summary of topic list and guiding questions used in focus group discussions

Topic:	Guiding question
Considerations for referral	What would you do in this case, and which patient and situational factors would you take into account in your decision-making?
Information needs for healthcare providers	What additional information would you need about the patient and/or the situation?
Patient preferences	How do you take patient and/or family preferences into account, and how do you explore these preferences?

Three focus groups were conducted to collect data. Previous research suggests that 80% of themes can be identified within three sessions [30]. The emergence of new themes was monitored, and no new themes appeared in the third focus group, indicating that thematic data saturation was reached. Baseline characteristics were collected, including age, sex, medical profession, years of work experience, and region of practice. Verbatim transcripts were produced by means of Microsoft Teams, after which all personally identifiable information was removed. The anonymised transcripts were subsequently stored on a secure server prior to analysis. The transcripts were not returned to the participants for feedback, and no repeat interviews took place.

### Data analysis

The focus group discussions were transcribed verbatim and analysed using the steps of the inductive thematic analysis approach: 1) familiarizing yourself with your data; 2) generating initial codes; 3) searching for themes; 4) reviewing themes; 5) mapping themes deductively based on four key consideration factors (patient-related, healthcare provider-related, case-specific, and structural considerations) from the literature [8] and 6) producing the report [31]. Transcripts were first openly and inductively coded. As the coding progressed, codes were compared, refined, and clustered into broader themes. First, the transcripts were analysed inductively through open coding. Subsequently, the resulting codes were analysed deductively by mapping them onto four key conceptual factors described in the literature: patient-related, healthcare provider-related, case-specific, and structural. While these factors provided a useful framework for organising and mapping our findings, the analysis remained open to themes that did not fit this structure. The transcripts were pseudonymized by removing directly identifying information and assigning pseudonyms to participants. The first focus group transcript was open coded line-by-line by the first and second researchers together (BW & CC) [25]. The remaining transcripts were coded by the two researchers independently (CC & BW). All steps of the analysis were iteratively developed through discussions between the two researchers (CC & BW). Additionally, key analytical discussions and interpretations were held until consensus was reached between authors (CC, BW, HM & LvE). Codes were iteratively clustered around the four key consideration factors until consensus was reached (CC & BW). The four key consideration factors, along with the codes identified in the analysis, were visualized in a figure to illustrate their influence and interactions with each other. Data analysis was performed using MAXQDA V24.

### Ethics

Our study was conducted in accordance with the Declaration of Helsinki [32]. The Medical Research Ethics Committee of Amsterdam University Medical Centre declared that the Medical Research Involving Human Subjects Act did not apply (registration number 2023.0479). When a healthcare provider expressed interest in participating, an email with information about the study was sent. At the start of each focus group, the moderator asked if the participants had any questions regarding their participation. All participants provided explicit verbal informed consent to participate in the study and consented to the audio-visual recording of the focus group. Participation in the focus groups posed no risks. All participants received a voucher worth 25 euros as a token of appreciation for their participation.

## Results

### Healthcare provider characteristics

In total, 16 participants took part in the study, divided across three focus groups (see Table 3). Three individuals intended to participate but withdrew shortly before the scheduled session due to personal or scheduling conflicts.

**Table 3:** Distribution of participants across focus groups sessions

**Variable**		**Focus group 1**	**Focus group 2**	**Focus group 3**	**Total (*N* =)**
Participants	Total participants	5	6	5	16
Profession in primary care	General practitioner (GP)	1		2	3
	Primary care nurse		1		1*
	Community nurse	1	3		4*
Profession in secondary care	Hospital nurse	1		1	2
	Nurse practitioner		1		1
	Physician assistant (PA)		1	1	2
	Medical doctor (MD)	2	1		3
	Medical resident			1	1

^*^One participant had two professions

Of the 16 participants, only one identified as male, while the others identified as female (Table 4). Participants represented both primary and secondary care roles: general practitioners (*N* = 3, 19%), primary care nurses (*N* = 1, 6%), a community nurse (*N* = 4, 25%), hospital nurses (*N* = 2, 12%), a nurse practitioner (*N* = 1, 6%), physician assistants (*N* = 2, 12%), medical doctors (*N* = 3, 19%), and a medical resident (*N* = 1, 6%). One participant held a dual role as both primary care nurse and a community nurse. Additionally, the participants had varying lengths of experience, ranging from 0 to 30 + years.

**Table 4:** Characteristics of study participants (*N* = 16)

Variable	Attribute	*N* = 16 (100%)
Sex	Male	*N* = 1
	Female	*N* = 15
Age	26 to 30 years	*N* = 6
	36 to 40 years	*N* = 3
	41 to 45 years	*N* = 1
	46 to 50 years	*N* = 1
	51 to 55 years	*N* = 1
	56 to 60 years	*N* = 3
	61 years or older	*N* = 1
Years of Experience	5 years or less	*N* = 7
	6 to 10 years	*N* = 4
	11 to 15 years	*N* = 1
	21 to 25 years	*N* = 2
	26 to 30 years	*N* = 1
	30 years or more	*N* = 1

^*^One participant had two professions

### Dynamic process of referral consideration

**Fig. 1:**
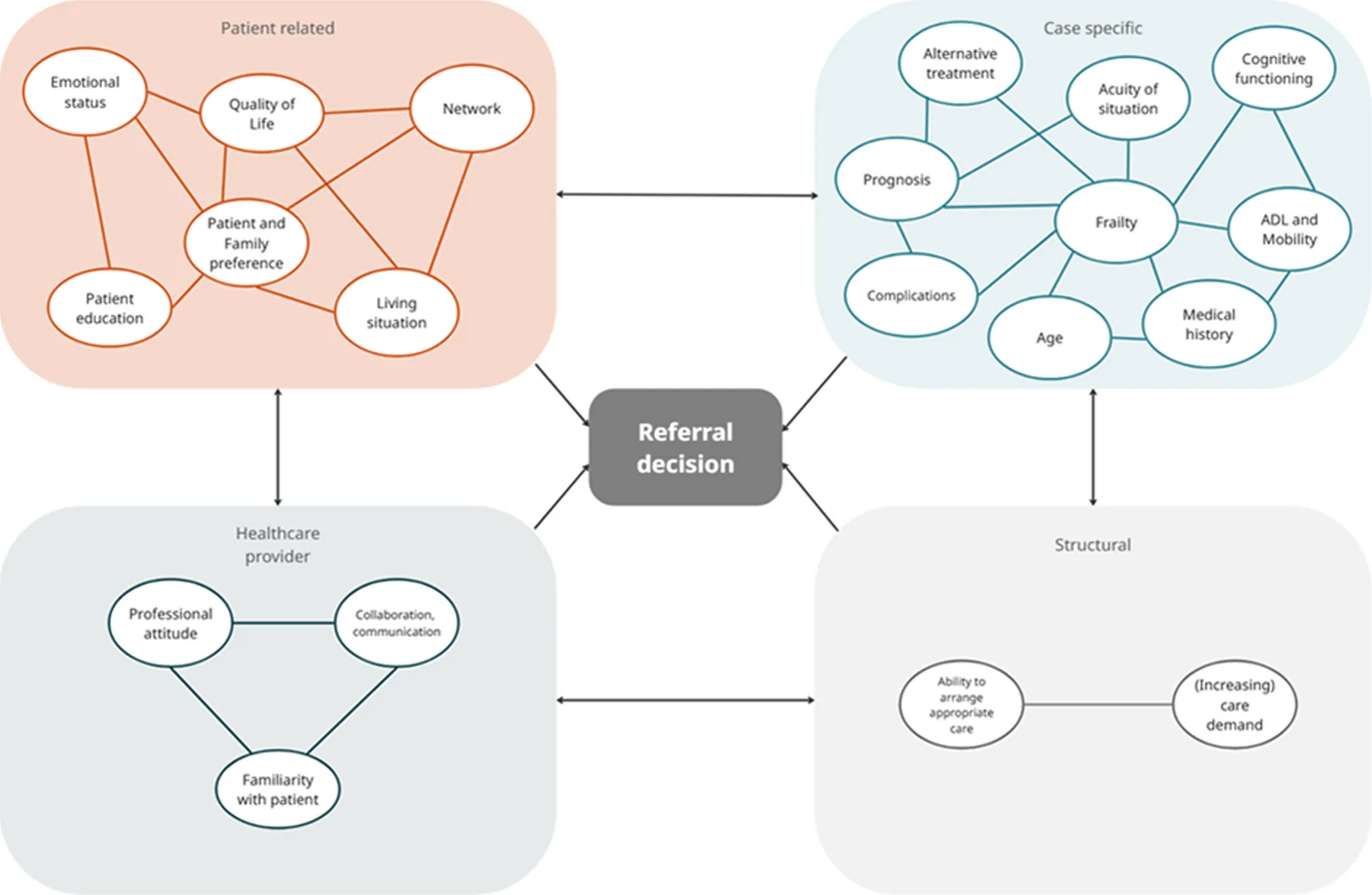
A conceptual synthesis of underlying considerations, structured around the four key consideration factors, that influence referral decisions. Legend: Circles represent the codes derived from the inductive thematic analysis of focus group data. Rounded rectangles depict the four key consideration factors, while the arrows illustrate interactions both within and between these factors, revealing a complex network of interrelated influences that shape the final referral decision

In total, 20 subthemes were identified inductively and deductively mapped into four overarching main themes, corresponding to the four key consideration factors. To illustrate the interrelated and dynamic nature of the considerations shaping referral decisions, these factors—patient related, case-specific, healthcare provider, and structural factors—are visually represented (see Fig. 1).

### Patient related consideration factors

The patient-associated considerations encompassed five subthemes, namely patient’s treatment preferences, emotional status, social network of the patient, quality of life, and living situation. An important patient-associated consideration factor was the *patient's treatment preferences.* Participants highlighted that ideally, ACP conversations about patient preferences should already have taken place. However, in the event of an acute situation, preferences of patients may change, as emphasized particularly by secondary healthcare providers. Therefore, participants believed that it is essential that patients and their families are still provided with clear and comprehensive information during acute situations. Participants emphasized the importance of enhancing *patient education* by clearly explaining the benefits and drawbacks of alternative treatments, such as pain management and palliative care. This would support well-informed decision-making and provide realistic expectations.

*"To gain more insight and overview, it's better to be well-informed, because at that age [and after a Hip surgery], you don't just bounce back out of bed"*—Hospital nurse.

*"People often don't realize they [may be] dying from something like a hip fracture – it all comes down to education and the time you invest. You [as a healthcare provider] have to paint the picture as clearly as possible for patients."* - MD

In addition, both primary and secondary healthcare providers highlighted the importance of family preferences. These preferences may conflict, thereby complicating the referral decisions of healthcare providers. The extent of the family's role and the weight of their opinions varied among healthcare providers. The role and influence of family preferences became even more critical in situations where patients lacked decision-making capacity, such as in cases of cognitive impairment. All healthcare providers agreed on the importance of involving the family in a timely manner to ensure alignment between the patient, family, and healthcare providers in acute situations.

*"If a patient is dominant in saying, [patient saying] “I don't want this anymore”, [but in some situations] that's almost impossible. Very often there are other family members who also have a lot of authority in the situation and who eventually say no, no, we are going to do this, or we want to do that. And then you see that it still happens."* - GP

Healthcare providers emphasized the importance of involving family in ACP discussions, ensuring that the family is aware of the patient’s preferences and that decisions are aligned, rather than coming as a surprise in the moment.

*"Of course, the patient is leading and not the children, but children also have to move on after their father's death. And if they feel we haven't done everything possible, you get a lot of processing problems. Apart from the anger you might get, the children should also be involved. So, if you make such an agreement, it means that you must make it clear in the ACP conversation that you really say, okay, are we going to write down that you won't be referred anymore? Is that really what you want, then we will write it down, but you can't just write it down, you also must discuss it with your children. And if you just write it down and then put it in the drawer, the children don't know that and they are put on the spot, so that's really not nice, so that can't happen."* – GP

*"But I do weigh the family more heavily than the patient, because I feel that the patient has lost insight into their illness." -* Primary care/community nurse

Another patient-associated consideration was the *emotional status* of the patient and family, particularly fear and uncertainty. For example, fear of stopping the treatment sometimes led patients to withhold information from healthcare providers.

*"They hide things from a doctor because they are afraid, they might not get the best treatment otherwise, or yes?"* - Hospital nurse

In such situations, it is important that healthcare providers engage in conversations with the patient and their family to provide reassurance and to clarify that alternative options are still available when curative treatment is no longer feasible. Additionally, participants emphasized the need to guide and support the family to assist in the process of acceptance, particularly in the event of the patient's impending death.

Participants also reported that emotions among family members, such as the desire of children to treat the patient as long as possible or anger, were important considerations in referral decisions.

*"Then I think I would refer, because indeed the GP also said apart from the anger the children might have towards you, it is especially very unpleasant for the children to have to live the rest of their lives with the idea that my father died because not everything was tried. Maybe he could have lived another six months and seen the birth of my child."* – MD

Furthermore, participants considered the *social network*, *living situation*, and degree of independence. Although the social network of patients, including informal caregivers, was often seen as a potential source of support, several healthcare providers emphasized its limitations. Especially single people with a small social network could not always rely on their network for care assistance. Additionally, participants highlighted that healthcare providers can become overburdened and need support, which is connected to broader care-related issues that also influence decision-making.

*"I think you can never do it right for everyone. And then maybe you should just protect the patient and make sure he doesn't get burdened with impossible care."* – PA

Finally, *quality of life* was also raised as a consideration factor in the decision to refer a patient to the hospital. Participants considered whether partners could remain together if the patient was referred to the hospital, whether visiting was possible, and whether the patient could still experience moments of happiness.

*"That gentleman really wants to stay with that lady; they have been together for a long time. And yes, you often see that one of the two is then admitted. And then they are still apart at the end of their lives."* - Primary care nurse

### Healthcare provider consideration factors

Considerations related to healthcare providers comprised three subthemes, which mainly revolved around their professional attitude, familiarity with their patients, and collaboration with other healthcare providers. *The professional attitude of healthcare providers* was a recurring theme. Participants indicated this included the use of personal experience and expertise in guiding decision-making and influencing patient choices, such as preventing adverse outcomes from surgery, sharing personal clinical experiences, experience with cultural background of the patients, and taking personal responsibility for patient care. Other aspects mentioned included the tendency to refer patients for convenience, shifting responsibility to other healthcare providers, assuming that other healthcare providers had already made the necessary considerations, and broader perspectives on healthcare challenges such as staff shortages and rising costs. However, the most prominent consideration across all participants was the healthcare providers’ intrinsic motivation to provide the best possible care and maintain a strong relationship with their patients.

*"If, as a doctor, you truly believe that a treatment is futile or even harmful – like if someone becomes delirious or develops pneumonia in the hospital – of course, you can steer the decision more strongly. But in a case like this, with a hip fracture and a competent, otherwise vital patient who might live another ten years..."* - GP

*"If I don't offer that option, I risk losing the patient relationship – and I think that relationship is essential, especially when such discussions need to take place."* - PA

Some participants from primary care experienced a barrier in discussing certain topics with secondary care providers. They indicated that they occasionally perceived blame when the other provider did not agree with the referral decision. In some cases, primary care providers referred patients more readily, shifting their responsibility to another provider, while secondary care providers were more cautious about the decision to refer patients.

*"I think we need to stop blaming each other. I've heard it myself when I was working in the hospital – GPs being blamed for patients ending up in the hospital beds. I used to think that sometimes too, but now I'm a GP, I see that everybody tries their best. We should stop pointing fingers and start working together."* – GP

Also, participants highlighted the importance of *familiarity with the patient*. When a provider is familiar with the patient's background and had established a therapeutic relationship, referral discussions were considered less complicated. Conversely, when patients were not well known, there was a greater tendency to refer.

*"This GP probably knows this man for 20 years. He's had the same GP all that time. That's the strength of having your own GP – you hope they'll be involved, because they already know how this man thinks and behaves. That makes having conversations [about their referral preferences] easier. Even if it's not documented, you've already had those conversations."* – GP

Secondary healthcare providers emphasized the added value of this familiarity with patients, expressing greater confidence in the assumption that primary healthcare providers had already made a well-considered decision in the referral process.

Participants also emphasized that *collaboration and effective communication between healthcare providers* can support the process of making complex care decisions. Participants noted that different providers often have varying types of therapeutic relationships with the patient. This diversity in perspectives contributes to a more holistic understanding of the patient and facilitates the delivery of optimal care. Moreover, participants noted that patients do not always share the same information with every healthcare provider. This underscores the importance of interprofessional communication.

*“It says that the pulmonologist essentially has no options left, but there’s no formal ‘no return’ policy in place. And in this case, an acute, not a maintenance situation—so those are important differences. The pulmonologist may not have any options left to improve the baseline with medication, but perhaps they can still contribute something in this acute exacerbation. It’s very complex.”* - Medical resident

*“In the hospital, I spend much more time with patients than a doctor does. A doctor might stop by for 5 to 10 minutes a day, but I’m with the patient for eight hours. That gives you a completely different picture. Sometimes you notice that a patient can present themselves as doing well for just those five minutes.”* - Hospital nurse

Differences in how well healthcare providers had a holistic view of the patient existed not only between primary and secondary care, but also within the same professional groups. Therefore, both primary and secondary healthcare providers emphasized the importance of effective information exchange. After the referral decision, a secondary healthcare provider indicated the importance of communication with the primary healthcare providers, to discuss the consideration underlying the referral.

### Structural consideration factors

The structural considerations encompassed two subthemes, which mainly concerned the *patient’s (increasing) care demand* and the *ability to organize appropriate care.* These aspects are interconnected in practice, as the nature and extent of a patient's care needs determine what type of care is required and whether it can be delivered. Participants described challenges related to the availability and organization of care, as well as the growing care demand of patients. Reported issues included the difficulty of finding a suitable care facility, the feasibility of remaining at home, the presence of rehabilitation indications such as the patient’s trainability, reducing the threshold for nursing home admission, and organizing 24-h home care. Participants also highlighted limitations within the healthcare system, such as the inability to scale up intensive or palliative home care, hospice care, or nursing home care. The increased burden on informal caregivers and the relative ease of arranging aftercare from within the hospital setting were also noted. Participants emphasized that these challenges are often more pronounced during evenings and nights.

*“[...] then the hospital physician ends up admitting the patient through the emergency department, or they’re admitted to a ward simply because there’s no aftercare available—and they’re just waiting there for a spot to open up*.”- MD

*"You really shouldn’t underestimate the care problem. It’s huge—even if someone has a large social network. Just operating a patient lift, for example...”* – GP

*"I think the biggest issue when deciding not to refer someone is how to organize the care."* – GP

All participants considered both the patient’s (increasing) care demand and the ability to arrange appropriate care in their decision-making, but the emphasis differed between primary and secondary care. For example, primary healthcare providers focused more on the challenges of organizing home care or nursing home care, with some mentioning that it was easier to arrange care once a patient was admitted to hospital. Secondary healthcare providers, on the other hand, were more concerned with the consequences of referring patients to the hospital without a clear medical indication, noting that patients often remain hospitalized unnecessarily while waiting for care arrangements.

### Case-specific consideration factors

All participants agreed on a diverse range of factors that comprise the case-specific considerations. Case-specific considerations varied across factors such as age, medical history, cognitive functioning, frailty, activities of daily living (ADL), mobility, acuity of the situation, prognosis, risk of complications and availability of alternative treatments.

Participants referred to the importance of *the patient's age and medical history, cognitive functioning*, and overall *frailty*. Medical history included an evaluation of the patient's care history, presence of comorbidities, such as cardiovascular disease, and medication use. Cognitive functioning includes cognitive problems such as dementia, the risk of delirium, and the degree of mental clarity and decisional capacity. Frailty included nutritional status, vitality, and signs of self-neglect.

*"He is very thin, he has few reserves, so if you sit for a long time, you naturally develop pressure sores, and eventually, you can get an infection from that."* – MD

Both a*ctivities of daily living (ADL)* and *mobility* were important considerations. These factors occurred both before and after the acute situation. For ADL, the focus was on estimating the patient's level of independence and whether they were already receiving assistance with ADL. Regarding mobility, the pre-acute situation involved determining whether the patient was able to mobilize independently, with or without aid. The participants took into account that if they did not refer the patient to the hospital, it was often assumed that the patient would become bedridden.

*"[From patients' perspective:] So I can't walk for the rest of my life. [If they say:] Fine, I'll just stay in bed at home and use a patient lift to move around. But it's such an abrupt transition – from one state to another.'".* - MD

Furthermore, the consideration of the *acuity of the situation* plays a role. If the patient is in an acute situation, decisions must be made immediately, which may differ from previously established wishes/choices. At that moment, there is no time to discuss this calmly, and it may result in different decisions being made in non-acute situations.

*“Decided in the moment, that is leading, you can't say, well, you said back then that you didn't want this and that, so goodbye”* – GP

Sometimes, the decision is made to admit patients during an acute situation in order to stabilize them before further evaluation.

“*In most acute situations, you still send someone to the hospital and then in calmer waters”* - Hospital nurse

Another consideration is assessing the *prognosis* of a patient. In the expected acute situation, the participants indicated that in COPD patients, the life expectancy is hard to predict. In the unexpected acute situation, this included the anticipated degree of recovery, taking into account possible complications, both with and without referral to the hospital.

*“I would also say that there are positive stories that people indeed get back on their feet quite quickly after surgery.” -* Medical resident

But also, the feasibility of returning home, estimating whether it is a short-term bridgeable problem or not, and the chance of death play a role. However, the prognosis is often difficult to predict, making the choice of whether or not to refer and treat difficult.

*“What you see is that the most obvious option is if people are not referred and you accept that they die within a foreseeable time”* – GP

A consideration in acute situations related to prognosis is the consideration of possible *complications*. This includes the healthcare provider's assessment of risks after a possible hospital admission/treatment, such as loneliness, delirium, infections, pneumonia, risk of death, fall risk, but also side effects of other treatment options, such as pain relief leading to drowsiness and confusion.

*“In this situation, I think I would send a bit more, I don't know if that's possible, but I want to emphasize that a hospital admission for her has a high chance of complications. And also, a higher chance of death, so you should clearly emphasize that compared to case one, right? That doesn't necessarily mean that they have a kind of prior chances.”* – GP

Both primary and secondary healthcare providers indicated that the risk of developing complications is a relevant consideration in referrals. While both sectors highlighted the risks of physical complications, one primary care provider also mentioned the risk of psychosocial complications, such as loneliness.

The prognosis and potential complications also influenced the choice of *alternative treatments*. Participants indicated that both healthcare providers and the patient and his or her loved ones should be informed about alternative treatment options. For patients with hip fractures, alternative approaches do not always require hospital admission. In such cases, patients may enter a palliative care trajectory focused on comfort and symptom management, including interventions such as pain blocks and morphine.

*“It really depends on the type of fracture, right? Whether it is indeed fairly easy to fix? I actually think that surgery itself is not even the biggest risk, because it almost always happens under local or spinal block or whatever that part. The point is actually that a fracture itself already causes a lot of disruption. And that's where the biggest risk actually lies*.” – GP

In the case of the COPD case, healthcare providers can also choose prednisone, bronchodilators, antibiotics, nebulization, etc.

*“This is very recognizable what you say that family just wants to continue treatment. Despite it being a case of a man with COPD and they feel that everything must be done. And well, that you are sometimes forced into certain actions and then go to the hospital, while you could have provided much better comfort at home.”* - Hospital nurse

But in both cases, if there are no curative treatment options left, palliative sedation or other non-therapeutic options can also be chosen.

*“If there are no treatment options left, can and should you as a medic also say that no more treatment will be given?”* - PA

However, each alternative treatment option has its own advantages and disadvantages. It is therefore important that healthcare providers communicate effectively with one another, such as through multidisciplinary team meetings, to discuss what is feasible and what is not.

*“Pain relief is also quite complicated, because she is a vulnerable woman, so she can easily become confused or drowsy from morphine patches or anything. So that's not easy either, so those pain blockades are very nice, but also not easy.” –* GP

Both primary and secondary healthcare providers indicated that the availability of alternative treatment options was a consideration in referral decisions. However, the expertise of the healthcare provider plays a role in this decision. For instance, some primary healthcare providers mentioned that for a patient to receive a pain block for a hip fracture, they must first be referred to the hospital. In contrast, some secondary healthcare providers noted that a pain block can also be administered in a home setting, making a hospital referral potentially unnecessary.

## Discussion

This study explored considerations of primary and secondary healthcare providers when deciding whether or not to refer older patients to the hospital in acute situations. Participants described a wide range of factors influencing decisions, which we organized into four key consideration factors: patient-related, healthcare provider-related, case-specific, and structural considerations. Our findings suggest that primary and secondary care providers may make different decisions regarding whether to refer, even in similar (acute) situations. Rather than being driven by a single factor, such decisions reflect a complex and dynamic interplay of multiple considerations. While a pilot study focused solely on GPs’ perspectives and other previous research has examined decision-making from the perspectives of patients and their families, our study adds value by including a wider range of healthcare providers, such as nurses, physician assistants, nurse practitioners and specialists [9, 33]. Although many referral considerations were shared between primary and secondary healthcare providers, differences in clinical backgrounds often led to varying considerations and ultimately to divergent decisions regarding whether or not to refer. This broader scope reveals tensions and mutual reinforcement between professional groups, showing that referral decisions are not only medically determined but also shaped by relational, contextual, and organizational factors. Important findings from our study are the importance of interprofessional communication and collaboration, the ability to arrange appropriate follow-up care, and the critical value of timely identification of patient and family preferences.

One key finding is the importance of strengthening interprofessional communication and collaboration between primary and secondary care providers, aligning with existing literature that identified such collaboration as essential for effective care coordination [34, 35] and for fostering peer support among healthcare providers [34]. Our results indicate possible variation in referral decision-making between primary and secondary healthcare providers, which may be influenced by differences in clinical backgrounds and information available about the patient. While both primary and secondary care providers aim to prevent inappropriate hospital transfers, their perspectives may result in varying referral outcomes. For example, focus group discussions revealed a discrepancy in awareness between primary and secondary care providers regarding hospital-level interventions that can also be delivered at home, such as home-administered pain blocks. This lack of awareness among primary care providers may contribute to a higher incidence of potentially avoidable hospital referrals. Furthermore, primary care providers often have better insight into external factors and patients' social contexts such as the patient’s living situation and social network, whereas secondary care providers rely on limited records, leading to potentially different referral decisions [36]. Therefore, primary care providers may refer patients to the hospital because of practical barriers, such as limited community care resources, lack of family support, or unfamiliarity with the patient, especially in the case of after-hours GP care [37]. In conclusion, our findings highlight the need for improved interprofessional communication and collaboration to ensure that healthcare providers share a common understanding of available care options, patient needs, and the broader social context influencing referral decisions, supported by accessible channels that enable healthcare providers to easily exchange information and ask questions [36, 37]. Practical strategies to strengthen such communication may include joint multidisciplinary meetings, structured feedback loops between primary and secondary care, and digital platforms for secure and timely exchange of patient information [38, 39].

Additionally, our study revealed that referral decisions are influenced not only by clinical background and knowledge of the patient’s situation, but also by the ability to arrange appropriate follow-up care. Our study identified several challenges in arranging appropriate care, including difficulties in finding suitable care facilities, assessing the feasibility of home-based care, evaluating rehabilitation potential, lowering the threshold for nursing home admission, and organizing 24-h home care. Primary care providers tended to focus on logistical barriers to arranging home or nursing home care, with some noting that care coordination was often easier once a patient was hospitalized. In contrast, and consistent with prior literature, our study demonstrated that secondary care providers expressed concerns about inappropriate hospital admissions without clear medical justification and prolonged hospital stays, often attributed to limited availability of appropriate care services or caregiver strain at home [40–44]. These differing perspectives highlight the need for better alignment and communication between primary and secondary care providers to support timely and appropriate referral decisions. So, fostering awareness of the shortages of the availability of appropriate patient care may strengthen mutual understanding among primary and secondary care providers. It may also support joint efforts to develop practical interventions for capacity constraints and healthcare organisation. This aligns with a recent study that describes care coordination as a negotiated process across organisational boundaries [45]. Importantly, our findings underline that structural constraints, such as capacity shortages in home care, hospice, or nursing homes, can at times outweigh medical considerations, effectively forcing referral decisions that may no align with patient preferences.

Finally, our findings highlight the critical value of timely ACP, including an early involvement of family members to help clarify patients’ care preferences before the onset of acute situations. In such situations, family members often play a pivotal role in the decision-making, acting not only as supporters but also as potential surrogate decision-makers [46]. However, literature has shown that surrogate decision-makers often misinterpret or inaccurately represent patients’ preferences, posing a significant risk to patient autonomy [47]. In practice, patients may clearly express a desire to avoid hospitalization, yet in acute situations this may change, or family members sometimes advocate for admission despite these wishes [48], thereby placing healthcare providers in ethically complex positions. Our study revealed a persistent tension between patient preferences and family influence, reflected in differing responses from healthcare providers. Some prioritized the patient’s stated preferences as the guiding principle, while others admitted patients despite their explicit wishes, citing strong family pressure during acute situations, as families often seek to pursue all available interventions in emotionally charged circumstances. Across all focus groups, participants highlighted the complexity of acute care decision-making, requiring the balance of patient preferences with familial expectations and emotional concerns. Involving family early in the ACP process helps to address the emotional burden of end-of-life decisions, which are sometimes perceived as actively ending life rather than allowing a natural death [49]. Early ACP discussions can therefore help ensure that care aligns with the patient’s values and goals and prevent ethical dilemmas for care providers. Although anticipatory approaches such as ACP are becoming more embedded in practice, they cannot fully resolve the complexity of acute referral decisions. ACP discussions are often challenged by the unpredictability of acute events and the emotional dynamics of patients and families in crisis. As a result, even well-prepared ACP documents may need to be revisited or contested during emergencies, underlining the importance of flexibility and continuous dialogue.

### Strengths and limitations

A key strength of our study is that the focus group cases and discussions revealed diverse and contrasting perspectives on referral decisions, highlighting the complexity, richness, and diversity of reasoning among primary and secondary healthcare providers that is also observed in practice. By including not only primary care providers but also secondary care providers, our study offers a more comprehensive and practice-relevant understanding of referral reasoning than would be possible from a single-provider perspective alone. This cross-disciplinary dialogue is particularly valuable in revealing different perspectives, tensions, and opportunities for improved coordination in referrals. Another strength of our study is the use of investigator triangulation. Focus groups were conducted and initially analysed by two researchers (CC & BW), followed by collaborative interpretation involving additional researchers (HM & LvE), with HM also present during one of the sessions. This approach enhanced the credibility and depth of the analysis by incorporating multiple perspectives and reducing individual bias [28, 50].

A first limitation of this study is the small and unevenly distributed sample (three focus groups, *N* = 16), which limits robust comparisons between professional groups. However, as the study was designed to be exploratory, any observed differences between primary and secondary care perspectives should be interpreted as tentative and hypothesis-generating rather than generalisable. Another limitation is that the presence of different types of healthcare providers may have led to hesitation in voicing critical opinions on other levels of care [50], potentially limiting the expression of critical views. However, differences in considerations between healthcare providers often emerged. This mixed composition created opportunities for mutual learning. Participants gained insight into the challenges faced by colleagues in other parts of the care continuum, and the exchange of perspectives fostered greater understanding and dialogue across professional boundaries. Another limitation concerns the order and diversity of the cases discussed. All focus groups followed the same sequence of cases, which may have influenced the depth of the focus group discussions. Typically, more time and energy were devoted to the first case, allowing participants to ease into the conversation, while the final case often received less attention due to time constraints. This imbalance may have affected the richness of insights generated for each case. In addition, a limitation relates to the content of the case vignettes used: two involved hip fracture and one involved COPD exacerbation. This may have shaped our findings. Other conditions (e.g. cognitive decline, infections, frailty) might have elicited different perspectives, and it remains unclear whether the identified domains are transferable across contexts. A more concrete and relevant example is cancer. As people live longer with incurable diseases, the prevalence of cancer is increasing worldwide, and cancer represents a distinct illness trajectory [51, 52]compared with conditions such as COPD (characterized by episodic decline) or hip fractures (typically acute). Due to the often intensive nature of the hospital-based cancer treatment, hospital-based providers frequently take on a different role compared to their involvement with patients with COPD or hip fracture and may therefore have different considerations regarding referral in acute situations. To obtain a more complete overview of considerations, including a cancer-related case could provide new insights into referral decision-making in the context of progressive, life-limiting illness.

### Recommendations and future research

A first recommendation is to strengthen interprofessional communication and collaboration between primary and secondary care providers. Accessible channels for information exchange and mutual consultation can help bridge differences in clinical background; facilitate the transfer of relevant patient information and preferences; increase awareness of available care options and help avoid unnecessary referrals. In addition, we recommend fostering awareness of the current shortages of appropriate care among both primary and secondary care providers, as this may enhance mutual understanding and facilitate collaborative efforts to develop practical strategies for managing these shortages, which are unlikely to be resolved in the short term. Finally, while family involvement is often already part of ACP, we recommend ensuring their active engagement throughout the process can help clarify patients' care preferences. This can foster shared understanding among all parties and reduce the likelihood of conflict or ethical uncertainty during acute decision-making.

Our findings should be interpreted within the Dutch healthcare context, where GPs act as strong gatekeepers. This organizational structure may shape how referral decisions emerge. For future research, a case study focusing on a different context, e.g., cancer, could provide valuable insights into whether similar or different considerations will occur in a context in which GPs and specialists have other care delivery responsibilities. Compared to more episodic conditions like COPD, cancer care typically involves greater specialist and multidisciplinary involvement, a more heterogeneous patient population, and different illness trajectories. Given its high prevalence and status as a leading cause of death, referral decision-making in this context is likely influenced by additional factors, thereby providing complementary insights beyond the current cases. In addition, incorporating the perspectives of patients and family members may offer insight into which referral considerations they find important and the challenges they experience in this process. Moreover, to enhance the alignment of care with patients’ preferences, we suggest that future research focuses on how interprofessional communication and collaboration can be improved in practice. It should also explore the feasibility and experiences of involving both patients and families, as well as the barriers and enablers that influence information transfer and collaborative decision-making across care settings.

## Conclusion and implications

This study identified the considerations that primary and secondary healthcare providers take into account regarding hospital referral decisions for older patients in acute situations. The findings suggest that such decisions are not determined solely by the acute condition but rather emerge from a complex and dynamic interplay of patient characteristics, healthcare provider-related factors, case-specific circumstances, and structural elements. Despite considerable overlap in the considerations across primary and secondary care providers, a variation in factors led to divergent referral decisions. The aim is therefore not to reach consensus on referral decisions, but to enhance mutual awareness of each other's perspectives and contexts. This shared awareness supports healthcare teams in making referral decisions that remain aligned with patient needs, while also taking into account care shortages and family dynamics. Enhancing mutual understanding and coordination between primary and secondary care providers, involving family early in the ACP process and addressing care shortages are therefore essential to support appropriate, patient-centred referrals. By recognizing and appreciating these diverse perspectives, care teams can better navigate complex decisions while ensuring that referral practices remain aligned with the needs and preferences of patients.

## Supplementary Information

Supplementary Material 1.

Supplementary Material 2.

Supplementary Material 3.

## Data Availability

The dataset used during the current study are available from the corresponding author on reasonable request.
