# Supplementary material for: Considerations in referring older patients to hospital in acute situations: a qualitative study based on the experiences of healthcare providers
Source: BMC Prim Care. 2026 Jul 20;27:331. doi: 10.1186/s12875-026-03453-9 (PMC13584258; doi:10.1186/s12875-026-03453-9)
Supplement: Supplementary file 2 [file 12875_2026_3453_MOESM2_ESM.docx]

**ADDITIONAL FILE 2: CASE DESCRIPTIONS**

**Case 1 Hip fracture**

Patient Jansen: 93-year-old man. He is a widower and live independently, performing activities of daily living (ADL) on his own. He receives housekeeping assistance once a week, and his son does his grocery shopping. The GP rarely sees him in the clinic, only for minor ailments occasionally and for the annual flu shot. He gives the GP the impression of a vital individual. Following his heart surgery in 2004, the GP already had a conversion about the end-of-life. He indicated that he wanted a do-not-resuscitate (DNR) and do-not-intubate (DNI) policy. These wishes are recorded in the Electronic Patient File and regularly re-discussed with him. Further Advanced Care Planning (ACP) wishes have not been discussed.

Medical history: Peripheral arterial disease (PAD), coronary artery bypass grafting (CABG) (2004).

Medication: Acenocoumarol, Pantoprazole, Temazepam as needed.

Situation: The patients' son calls the GP, reported that his father fell during the night. He was able to get up on his own and lay down on the bed, but experienced immediate severe pain. He did not want to contact the GP out-of-hours service and took paracetamol. Now, the pain is so intense that he is unable to get out of bed. His son went straight to his father and found him lying in bed. The patient has severe pain in his right groin, buttock, and thigh. The GP visits him immediately after the morning consultation.

Upon arrival, the GP finds a painful, alert, and adequate patient with normal vital signs. The GP immediately notices the right foot is in exorotation. The pain in the right buttock is so severe that an examination of the leg and hip joint is not possible. The working diagnosis is a hip fracture.

**Case 2 Hip fracture**

Patient de Boer: 90-year-old woman. She lives with her 89-year-old husband in an apartment on the ground floor. They have no children. She has Alzheimer's disease and receives daily home care for dressing and undressing. She can move around the house herself with a walker. Meals are provided by a meal service. Her husband does other shopping himself and there is housekeeping assistance once a week. The husband is mobile. He has a pacemaker but otherwise has few health issues. The dementia case manager is closely involved with this couple. The GP has already discussed several times with the patient, her husband and the case manager that it is a delicate balance because a lot rests on her husband's shoulders. In particular, the toilet visits and sometimes night-times restlessness of the patient demand a lot of the husband. He absolutely does not want his wife to be admitted to a nursing home. The GP had a conversation about the end of life with both of them and both indicated that a do-not-resuscitate (DNR) and do-not-intubate (DNI) policy.

Medical history: Alzheimer's disease, diabetes mellitus

Medication: Rivastigmine, Omeprazole, Lisinopril

Situation: Her husband calls the emergency line of the GP in the afternoon. The patient has fallen and can no longer get up; he cannot get her up. She has severe pain in her lower back and left hip. The GP immediately goes on an emergency visit.

Upon arrival, the patient is lying on the floor. She reports a severe pain in her left hip and seems anxious. During her physical examination, her vitals are fine. The left leg is in exorotation and appears shortened. The GP suspects a hip fracture.

**Case 3 exacerbation of the COPD**

Patient de Vries: 78-year-old man, lives with his 75-year-old wife in a single-family home. He has very severe COPD (GOLD-IV). He receives maintenance medication from the pulmonologist and uses chronic oxygen. Daughter and son live nearby and are closely involved. The wife does almost everything in the house. Their daughter only helps with the housekeeping. He has been sleeping in a high-low bed in the living room for one year because he can no longer climb the stairs. A stair lift is not possible for the stairs in his house. His regular pulmonologist has indicated that there are actually no more therapeutic options that the hospital can offer him. The children in particular are quite urgent in their request for admission when the patient is short of breath. The patient himself actually does not want to go to the hospital anymore but has indicated that it would be too difficult for his children, because they would like the man to be treated as intensively as possible. His wife does not want to make a decision about it and leaves the decision about whether or not to admit him to the man. The GP had an Advanced Care Planning (ACP) meeting after the last admission and the patient indicated that he had a no resuscitation, and no intubation wish and also did not want any more ICU admissions. The children find this wish difficult.

Medical history: COPD GOLD-IV, diabetes mellitus, myocardial infarction (2010), chronic heart failure.

Medication: Trimbow (Beclomethasone/Formpterol/Glycopyrronium), Salbutamol, Azithromycin, Prednisolone in maintenance dosage, Simvastatin, Hydrochlorothiazide, Lisinopril, Ascal

Situation: The wife calls: the patient has been very short of breath again since last night, the inhalation medication does not help, even by increasing the oxygen a little they cannot get the shortness of breath under control. The GP suspects an exacerbation of COPD.
