# Supplementary material for: Considerations in referring older patients to hospital in acute situations: a qualitative study based on the experiences of healthcare providers
Source: BMC Prim Care. 2026 Jul 20;27:331. doi: 10.1186/s12875-026-03453-9 (PMC13584258; doi:10.1186/s12875-026-03453-9)
Supplement: Supplementary file 3 [file 12875_2026_3453_MOESM3_ESM.docx]

**ADDITIONAL FILE 3: Interview Guide**

**1. Introduction**

- Welcome
- Introducing researchers
- Aim of study
- Explanation of how the meeting will proceed and what participants can expect.
- Instruction online participation (microphone, video, chat use, notes taken during meeting etc.)
- Audio and video recording
- Informed consent
- Questions

**2. Case studies**

Characteristics and information needs

Presenting the fictive patient case study:

- Each healthcare provider notes for themselves:
  - What they would do in this case and which characteristics of the patient and the situation they consider in their decision-making.
  - Their assessment of the likely outcome of the continuation of this case.
  - What additional information they would like to have (about the patient and/or the situation).
- The considerations, assessments of the outcome, and information needs are further explained.
- The facilitator highlights the differences and similarities regarding the considerations, asking healthcare providers to reflect on these differences. Questions such as: "Could you elaborate on that?", "What exactly do you mean?", and "Could you provide an example?" may be used to guide the conversation.
- The facilitator also points out the differences and similarities in the information needs, prompting the group to collaboratively prioritize these information needs.

Conversation with patient about their preferences

- If information needs related to the patient's preferences are mentioned: How would you gather this information? In the case of a conversation, ideally, when would you have this discussion with the patient?
- If patient preferences are not mentioned: How would you take the patient's preferences/goals (life perspective) into account? Which preferences specifically? How would you uncover these? And when would they ideally be discussed? What if your considerations differ from the patient's wishes/preferences?

Information provision to patient

- Regarding the above considerations in the case, what role does the patient themselves, and their relatives/family, play in the decision-making process?
- The information that each healthcare provider would provide is shared and further explained.
- Given all the information, what is most and least important for a patient to know?
- Are there situations or specific patients where you would provide no information or offer different information?

**3. Reflection**

Characteristics and Information Needs

- There is reflection on the findings of all the discussed cases, highlighting differences and similarities between them. The group is asked for any explanations or reasons for these differences.
- Overall, what role do healthcare providers see for themselves in such scenarios? If a healthcare provider does not see a (major) role for themselves, which other healthcare professionals do have a role?

Discussion with the Patient about Preferences

- What is the core information a healthcare provider should know about a patient in light of the considerations discussed?

Information Provision to the Patient

- In light of the discussed cases, what information would patients need in order to make well-informed decisions?
- Is there a need for additional support in informing patients (tools, personal skills)?
- The discussion has been based on an ideal situation, but what barriers do general practitioners see in translating this ideal situation into practice?
- What potential solutions exist for these barriers?

**4. Closure**

- A brief reflection from the researchers on what has been discussed in the focus group
- Additional insights?
- Stop recording.
